# Record‐High Ultrasound‐Sensitive NO Nanogenerators for Cascade Tumor Pyroptosis and Immunotherapy

**DOI:** 10.1002/advs.202302278

**Published:** 2023-07-03

**Authors:** Yuheng Bao, Yanni Ge, Mengjie Wu, Zhengwei Mao, Juan Ye, Weijun Tong

**Affiliations:** ^1^ MOE Key Laboratory of Macromolecular Synthesis and Functionalization Ministry of Education Department of Polymer Science and Engineering Zhejiang University Hangzhou Zhejiang 310027 China; ^2^ Eye Center The Second Affiliated Hospital School of Medicine Zhejiang University Zhejiang Provincial Key Laboratory of Ophthalmology Zhejiang Provincial Clinical Research Center for Eye Diseases Zhejiang Provincial Engineering Institute on Eye Diseases Hangzhou Zhejiang 310009 China; ^3^ Stomatology Hospital School of Stomatology Zhejiang University School of Medicine Zhejiang Provincial Clinical Research Center for Oral Diseases Key Laboratory of Oral Biomedical Research of Zhejiang Province Cancer Center of Zhejiang University Hangzhou Zhejiang 310058 China

**Keywords:** cascade tumor therapy, cGAS‐STING, hollow MnO_2_, NO donor, pyroptosis, ultrasound response

## Abstract

Pyroptosis is a pro‐inflammatory cell death that is associated with innate immunity promotion against tumors. Excess nitric oxide (NO)‐triggered nitric stress has potential to induce pyroptosis, but the precise delivery of NO is challenging. Ultrasound (US)‐responsive NO production has dominant priority due to its deep penetration, low side effects, noninvasion, and local activation manner. In this work, US‐sensitive NO donor *N*‐methyl‐*N*‐nitrosoaniline (NMA) with thermodynamically favorable structure is selected and loaded into hyaluronic acid (HA)‐modified hollow manganese dioxide nanoparticles (hMnO_2_ NPs) to fabricate hMnO_2_@HA@NMA (MHN) nanogenerators (NGs). The obtained NGs have a record‐high NO generation efficiency under US irradiation and can release Mn^2+^ after targeting the tumor sites. Later on, cascade tumor pyroptosis and cyclic GMP‐AMP synthase‐stimulator of interferon genes (cGAS‐STING)‐based immunotherapy is achieved and tumor growth is effectively inhibited.

## Graphical abstract

Cascade tumor pyroptosis and cyclic GMP‐AMP synthase‐stimulator of interferon genes (cGAS‐STING) immunotherapy at tumor sites by hMnO_2_@HA@NMA (MHN) nanogenerators (NGs). MHN NGs can produce nitric oxide (NO) under ultrasound irradiation and release Mn^2+^ ion. After pyroptosis induced by NO, double‐stranded DNA is released from tumor cells, which contributes to Mn^2+^‐enhanced cGAS‐STING pathway.

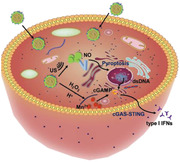

## 1 Introduction

Due to its high incidence and mortality, tumor has been regarded as the leading cause of death among human beings.^[^ ^1^ ^]^ Pyroptosis, other than apoptosis and necrosis, is a pro‐inflammatory programmed cell death which has raised interest among tumor therapies in recent years.^[^ ^2^ ^]^ In its inflammatory microenvironment, it is more likely to offer great contributions to activate innate immunity against tumors.^[^ ^3^ ^]^ Nitric oxide (NO) is a gaseous molecule, which functions as an important signaling transductor in metabolism systems, such as nervous system,^[^ ^4^ ^]^ cardiovascular system,^[^ ^5^ ^]^ and immune system.^[^ ^6^ ^]^ NO maintains relatively low level in organisms, but the local surging of NO concentration may raise nitric stress and induce cellular pyroptosis,^[^ ^7^ ^]^ which has been used as a novel target for tumor intervention.

However, the delivery of NO is hindered by its poor stability in vivo.^[^ ^8^ ^]^ A switchable NO donor is required for precisely controlled release of NO in tumor sites. Exogenous stimuli have been utilized for NO release, such as light^[^ ^9^ ^]^ and X‐ray,^[^ ^10^ ^]^ for their highly directional ability for local controllable NO release. Among all exogenous methods, ultrasound (US) has dominant priority for its deep penetration, low side effects, and completely noninvasive treatment.^[^ ^11^ ^]^ Thus, efficient US‐triggered NO donor is raised as concern in NO‐based tumor therapeutic methods.

L‐arginine (LA) is an endogenous NO donor, but its highly hydrophilic nature^[^ ^12^ ^]^ and the essential substrate of nitric oxide synthase (NOS)^[^ ^13^ ^]^ restrict the NO production precisely in tumors. N‐Nitrosamines, produced in stomach, are chemically stable pools of NO molecule, which can be activated by exogenous stimuli to release NO.^[^ ^14^ ^]^ Up to now, *N*,*N*′‐Di‐sec‐butyl‐*N*,*N*′‐dinitroso‐1,4‐phenylene diamine (BNN6) has been reported for targeted US‐based NO production,^[^ ^15^ ^]^ but the NO production property still needs to be enhanced through optimizing the structure of *N*‐nitrosamines. Band gap theory has been widely used in sonosensitizer design, and narrowing the bandgap of US‐responsive molecules has positive effects on their stimuli‐activation abilities.^[^ ^16^ ^]^ Thus, efficient NO donors can be discovered by designs of their molecular bandgaps.

Efficient NO donors need to be delivered to target region, and different nanomaterials have been widely explored as carriers for their delivery to tumor sites.^[^ ^17^ ^]^ Manganese oxide nanomaterials have broad applications in biomaterials because of their tunable structures, unique catalytical properties, and good biosafety.^[^ ^18^ ^]^ Among these nanomaterials, manganese dioxide nanoparticles (MnO_2_ NPs) have been chosen as a catalase‐like nanozyme to decompose H_2_O_2_ into oxygen and water.^[^ ^19^ ^]^ This catalytical property of MnO_2_ NPs allows them to be used as effective oxidative stress scavengers for anti‐inflammation^[^ ^20^ ^]^ and hypoxic environment alleviators^[^ ^21^ ^]^ for tumor therapies. Our previous work utilized hollow MnO_2_ nanozymes as carriers to deliver budesonide for synergistic inflammatory bowel disease therapy.^[^ ^22^ ^]^ The process of catalyzing H_2_O_2_ can simultaneously release Mn^2+^, contributing to cyclic GMP‐AMP (cGAMP) formation caused by pyroptosis‐induced double‐stranded DNA (dsDNA) release for subsequent tumor cGAS‐STING (cyclic GMP‐AMP synthase‐stimulator of interferon genes) immunotherapy.^[^ ^23^ ^]^

**Scheme 1:**
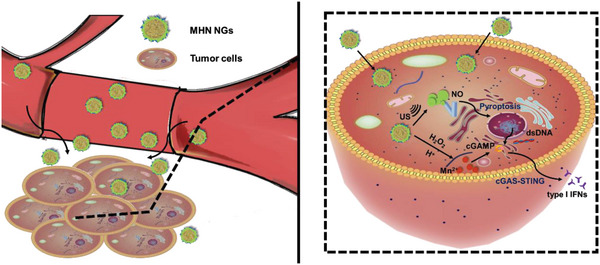
Schematic illustration of the biological mechanism of MHN NGs in circulation and the subsequent cascade tumor pyroptosis and cGAS‐STING immunotherapy therapy at tumor sites.

Herein, NO donor *N*‐methyl‐*N*‐nitrosoaniline (NMA) with designed structure was first selected according to the band gap theory for US‐triggered NO production. After NMA was loaded into hyaluronic acid (HA)‐modified hollow MnO_2_ nanoparticles (hMnO_2_ NPs), the obtained hMnO_2_@HA@NMA (MHN) nanogenerators (NGs) were able to accumulate at tumor sites through CD44 active targeting, and release Mn^2+^ ions in the tumor microenvironment (TME). MHN NGs functioned as a US‐responsive NO generator to locally release NO and induce tumor pyroptosis. Augmented by Mn^2+^ ion, the released cellular dsDNA contributed to cascade activation of cGAS‐STING pathway for subsequent immunotherapy (**Scheme** **1**).

## 2 Results and Discussion

### 2.1 NO Donor Selection

*N*‐Nitrosamines are one type of reactive organic molecules with the chemical structure of R_2_N—N=O, and the N—N single bond can be cleaved by the attacking of free radicals to form gaseous NO or •NO radicals.^[^ ^24^ ^]^ US irradiation has the potential for abundant free radical production, thus contributing to N—N bond destruction and NO production.^[^ ^25^ ^]^ According to the bandgap theory, the singly occupied molecular orbital (SOMO)‐lowest unoccupied molecular orbital energy of molecules is closely connected with the reaction accessibility during the nucleophilic radical reaction thermodynamically.^[^ ^26^ ^]^ Thus, density functional theory (DFT) was used to simulate the structures of BNN6 and NMA for their SOMO energy levels (Figure S1A–D, Supporting Information). In the case of double *N*‐nitrosamine group in BNN6, its SOMO energy was −5.709 eV, lower than that of NMA (−5.669 eV) with single *N*‐nitrosamine group. The higher SOMO energy level of NMA indicated the better accessibility for nucleophilic radical reaction compared with that of BNN6,^[^ ^27^ ^]^ resulting in the higher reactivity of NMA to produce NO with US irradiation.

**Figure 1:**
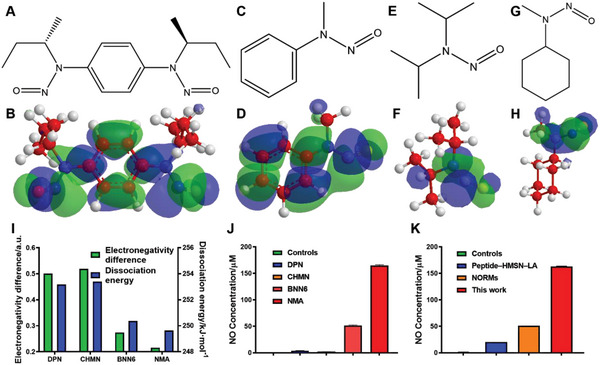
Chemical structure and schematic illustration of negative (green) and positive (blue) charge center of A,B) BNN6, C,D) NMA, E,F) DPN, and G,H) CHMN molecules with Chem3D. I) Electronegativity difference and dissociation energy of N—N bond in DPN, CHMN, BNN6, and NMA molecules by DFT simulation with AMSjobs. J) NO production by MH encapsulated DPN, CHMN, BNN6, and NMA (*n* = 3). K) The NO production property of MHN NGs in this work compared with peptide‐HMSN‐LA^[^ ^13a^ ^]^ and NORMs^[^ ^15a^ ^]^ in previous reports (*n* = 3).

Apart from the free radical reactivity, the stability of N—N single bond is also a crucial factor in this reaction. We first used 3D Chem to minimize the molecular energy, and the schematic illustration of electron cloud distributions of BNN6 (**Figure** **1**A,B) and NMA (Figure 1C,D) is obtained. Despite the aforementioned NO donors, *N*‐nitrosamine‐type NO donor *N*,*N*‐diisopropylnitrous amide (DPN, Figure 1E,F) and *N*‐cyclohexyl‐*N*‐methylnitrous amide (CHMN, Figure 1G,H) were also investigated with the same simulation approach. The electron cloud distribution of atoms in both ends of N—N bond varied more narrowly in NMA than that of other donors, which led to a less stable N—N bond in NMA molecule that was easier for bond breakage.^[^ ^28^ ^]^ To be quantified, the electronegativity difference of N—N bond in NMA was 0.215, lowest in aforementioned molecules (Figure 1I). To further prove this conclusion, the molecular bond dissociation energy of above NO donors was calculated after simulation.^[^ ^29^ ^]^ The bond dissociation energy of N—N bond in NMA was 249.64 kJ mol^−1^, less than other NO donors, which was the evidence for its easiest bond breakage and the best NO production property. Based on the above simulated results, NMA may have the best NO production property among these *N*‐nitrosamine‐type NO donors.

After molecular simulation, equal concentration of DPN, CHMN, BNN6 and NMA molecules was respectively treated with US, and the NO production activity was measured with NO assay kit (Figure 1J). NMA showed the best NO production activity, conforming to the results of simulation. Later, the NO production property of NMA was also compared with the results in previous reports (Figure 1K). After normalized process, NMA molecule showed three times NO production property as high as that of NORMs,^[^ ^15a^ ^]^ and seven times higher than that of peptide‐HMSN‐LA.^[^ ^13a^ ^]^ These results confirmed the record‐high NO production property of NMA due to the optimized structural design.

### 2.2 Preparation and Characterizations of MHN NGs

**Figure 2:**
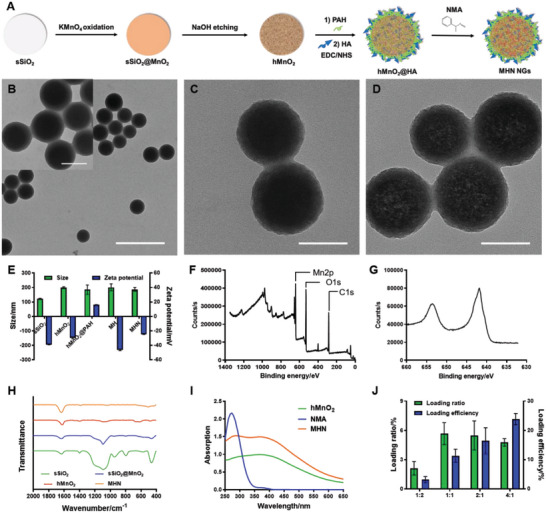
The synthetic procedure and characterization of MHN NGs. A) The schematic illustration of synthesis route of MHN NGs. B) TEM images of sSiO_2_, scale bar: 500 nm (amplified image on the left upper corner, scale bar: 100 nm); TEM images of C) sSiO_2_@MnO_2_ and D) MHN NGs, scale bar: 100 nm. E) Particle size and zeta potential variation during synthesis (*n* = 3). XPS F) full spectrum scanning analysis and G) fine spectra scanning of Mn 2p of MHN NGs. H) IR spectra of sSiO_2_, sSiO_2_@MnO_2_, hMnO_2_, and MHN NGs. I) UV‐vis spectra of hMnO_2_, NMA, and MHN NGs and J) NMA loading properties (*n* = 3).

Solid SiO_2_ nanospheres (sSiO_2_) were used as templates for the preparation of hMnO_2_ NPs. KMnO_4_ solution functioned as the oxidant and the Mn donor is used for the in situ growth of MnO_2_ on the surface of sSiO_2_, thus sSiO_2_@MnO_2_ NPs were formed. Then, the templates were removed by adding NaOH solution and hMnO_2_ NPs were synthesized. After the assembly of poly(allylamine) (PAH) and HA on their surface, NMA was finally loaded into HA‐modified hMnO_2_ NPs (MH NPs) to obtain the ultimate MHN NGs (**Figure** **2**A). Compared with sSiO_2_ and sSiO_2_@MnO_2_ NPs, MHN NGs showed abundant pores inside with an average size of 12.67 nm (Figure 2B–D and Figure S2, Supporting Information), resulting from the hollow nanostructure of hMnO_2_ NPs. Dynamic light scattering analysis was conducted throughout the synthetic procedure (Figure 2E). The size of hMnO_2_ NPs slightly increased after MnO_2_ layer was formed, and then kept stable during the process of surface modification and NMA loading. The size of finally obtained MHN NGs was 186.5 ± 10.4 nm. The alternative reversal of surface zeta potential of the NPs during the assembly of PAH and HA (Figure 2E) also confirmed the successful modification of HA, which can endow the NPs with the targeting ability to CD44.^[^ ^30^ ^]^

To verify the detailed chemical structure of MHN NGs, X‐ray photoelectron spectroscopy (XPS) analysis was first used to determine the elemental composition of the obtained NPs (Figure 2F and Figure S3A,B, Supporting Information). The peaks of C 1s, O 1s, and Mn 2p were clearly observed, which proved the abundant presence of C, O, and Mn elements in MHN NGs. Moreover, compared with the standard MnO_2_ binding energy in NIST XPS database (Mn 2p_1/2_: 653.8 eV, Mn 2p_3/2_: 642.2 eV), the peaks of synthesized MHN NGs conformed to the spectral line of MnO_2_ crystal (Figure 2G).^[^ ^31^ ^]^ Further IR spectrum analysis showed the disappearance of Si—O—Si antisymmetric stretch wide peak at 1095 cm^−1^ after alkali etching, which suggested the complete removal of SiO_2_ template (Figure 2H). The above two results were better proved by X‐ray diffraction analysis checked with standard SiO_2_ (PDF#29‐0085)^[^ ^32^ ^]^ and MnO_2_ (PDF#44‐0992)^[^ ^33^ ^]^ (Figure S4, Supporting Information).

Later, UV‐vis spectra were used to prove the composition of MnO_2_ and NMA in the final MHN NG products (Figure 2I). The absorption curves showed the presence of both hMnO_2_ and NMA. To study the best loading condition for NMA, UV‐vis spectra were used to evaluate the loading ratio and loading efficiency of NMA in MHN NGs with different feeding ratio (Figure 2J). We chose 1:2, 1:1, 2:1, and 4:1 as the feeding ratio (MH/NMA, mass/mass), and then the ultimate loading properties were measured. After the standard absorption curve in ethanol was produced (Figure S5A, Supporting Information), the loading properties of NMA molecule were obtained. After the feeding ratio reached 1:1, the loading ratio exhibited a plateau, so we chose 1:1 as the optimized feeding ratio. In this condition, MHN NGs had the highest loading ratio of 5.66 ± 1.20%, and the loading efficiency of NMA was 11.33 ± 2.40%.

### 2.3 In Vitro Responsive Release, NO Production, and Stability

**Figure 3:**
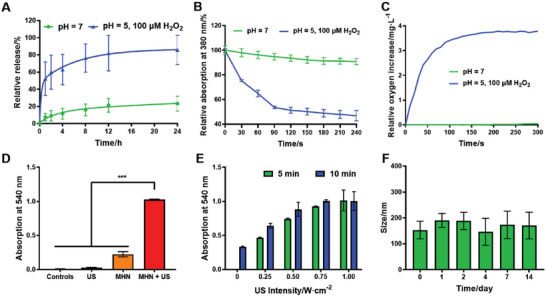
In vitro TME‐responsive release, NO production properties, and stability of MHN NGs. A) NMA release properties in neutral and acidic H_2_O_2_ environment (*n* = 3). B) Degradation property of MHN NGs monitored with UV‐vis spectra (*n* = 3) and C) dissolved oxygen analysis. D,E) In vitro US‐triggered NO production by MHN NGs with NO assay kit (*n* = 3, ***: *p* < 0.001). F) Size stability analysis of MHN NGs in DMEM/FBS solution for 14 days (*n* = 3).

The release curves of MHN NGs in neutral and tumor‐mimicking environment were studied (**Figure** **3**A and Figure S5B, Supporting Information). A relatively quicker releasing rate of NMA was observed, with nearly 50% of total NMA amount released in only 1 h. This quick release rate of hydrophobic molecule was particular, which was due to the degradation property of MHN NGs in acidic TME with high level H_2_O_2_ (form. 1). In this mimicking environment, MHN NGs presented a higher degradation rate than that in neutral environment, which was greatly higher than similar Mn‐based NPs in previous reports (Figure 3B).^[^ ^34^ ^]^ This may result from the highly amorphous and hollow structure of hMnO_2_ that was easier to be etched (Figure S4, Supporting Information). MHN NGs can be catalyzed by acidic H_2_O_2_ in TME, and then Mn^2+^ ion and oxygen were produced from these NGs. The production of oxygen at this condition by dissolved oxygen analysis gave a persuasive clue to clarify this reaction phenomenon (Figure 3C). The reaction process can be described as the following formula

(1) $${\mathrm{MnO}}_{2}+H_{2}O_{2}+2H^{+}={\mathrm{Mn}}^{2+}+2H_{2}O+O_{2}↑$$

Apart from quick release of NMA in tumor sites, the degradation of MHN NGs also has the benefit to locally release Mn^2+^ ion at tumor sites. As is reported in previous researches, the release of Mn^2+^ ion has the potential to enhance tumor immunotherapy through cGAS‐STING pathway.^[^ ^35^ ^]^ Released Mn^2+^ ion can also induced in vitro •OH production by Fenton‐like reaction (Figure S6, Supporting Information). Therefore, MHN NGs in this work can simultaneously serve as a NO donor carrier, and also an immune agonist for subsequent tumor immunotherapy.

The in vitro NO production of MHN NGs under US irradiation was monitored by NO assay kit (Figure 3D). Compared with bare US or MHN NGs group, MHN NGs irradiated with US showed a very significant NO production increase (*p* < 0.001), which proved that MHN NGs have the capacity of producing abundant NO in solution. The relation between irradiation conditions of US and NO production was also studied (Figure 3E). The NO amount increased with the augmented US intensity and time, and the maximum value was obtained after US intensity reached 1 W cm^−2^.

To study the in vitro stability, MHN NGs were added to Dulbecco's modified Eagle medium (DMEM)/10% fetal bovine serum (FBS) cell culture medium for 14 days (Figure 3F). The size of MHN NGs kept steady in the measurement period, which greatly verified that the HA‐camouflaged MHN NGs were capable to maintain the colloidal stability in biological environment.

### 2.4 Cellular Uptake and Cytotoxicity

**Figure 4:**
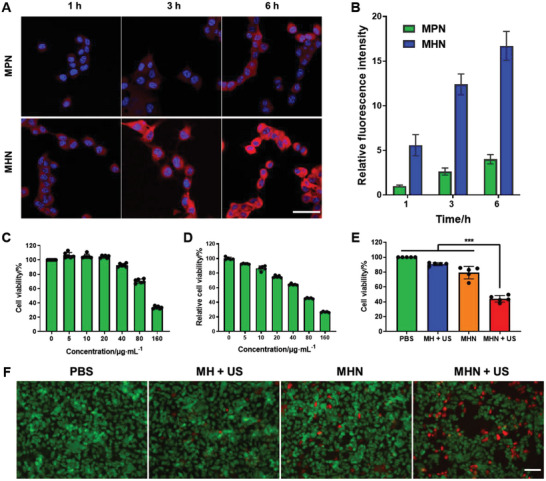
Cellular uptake and cytotoxicity studies of MHN NGs. MPN NGs and MHN NGs were dyed and co‐incubated with B16F10 cells for 1, 3, and 6 h. A) The intracellular fluorescence images were observed by confocal microscopy (scale bar: 50 µm). B) Statistical analysis of intracellular fluorescence intensity measured by flow cytometry (*n* = 3). Cellular toxicity of MHN NGs after incubation for 24 h with C) 3T3 cells and D) B16F10 cells at different concentrations (*n* = 5, ***: *p* < 0.001). E) B16F10 cell viability analyzed by CCK‐8 (*n* = 5) and F) live–dead staining with calcein AM (green) and propidium (red) after treated with HMN NGs and US, scale bar: 100 µm.

To determine the cellular uptake property of MHN NGs, MP@NMA (MPN) NGs were synthesized by replacing HA with poly(acrylic acid) (PAA) from MHN NGs. Then, MPN NGs and MHN NGs were dyed with Nile Red and co‐incubated with B16F10 cells. After incubation for 1, 3, and 6 h, the confocal images of both groups were captured (**Figure** **4**A). To quantitatively analyze the cellular uptake effects, the intracellular fluorescent intensity was also measured by flow cytometry (Figure 4B). The cellular uptake of MPN NGs and MHN NGs gradually increased during 6 h. The fluorescent intensity of MHN NGs was much stronger than that of MPN NGs. Besides, the MHN NGs were also incubated with 3T3 cells (normal cell line) and B16F10 cells. The endocytosis efficiency of B16F10 was much higher than that of 3T3 cells (Figure S7, Supporting Information), which resulted from the specific binding between the CD44 on cancer cells and the HA on MHN NPs.

The cytotoxicity of MHN NGs was conducted by 3T3 cells (normal cell line, Figure 4C) and B16F10 cells (tumor cell line, Figure 4D). Different concentrations (0, 5, 10, 20, 40, 80, and 160 µg mL^−1^) of MHN NGs were, respectively, cultured with these two cells, and the cell viability was obtained by cell counting kit‐8 (CCK‐8) after 24 h. MHN NGs maintained low cytotoxicity for normal cells below 40 µg mL^−1^, but the cytotoxicity of MHN NGs for tumor cells was higher than that of normal cells. The augmented toxicity of MHN NGs for tumor cells may be attributed to higher uptake efficiency of MHN NGs by tumor cells caused by upregulated CD44 expression in tumors. Also, the release of Mn ion from MHN NGs in the relatively acidic TME compared with normal cells can damage cells by Fenton‐like reaction. This hypothesis was proved by in vitro •OH production with cellular reactive oxygen species (ROS) probe (Figure S8, Supporting Information).

Later, the therapeutic effect of MHN NGs under US irradiation was tested with B16F10 cells. Moderate US irradiation intensity was first chosen for cellular studies to avoid obvious cellular toxicity (Figure S9, Supporting Information). Cells were divided into four groups, and were, respectively, treated with phosphate‐buffered saline (PBS), MH + US, MHN, and MHN + US. While the US‐treated MH NGs group remained high cell viability (over 80%), the US‐treated MHN NGs group exhibited very significant tumor cell inhibition effect (Figure 4E, *p* < 0.001). Although MHN NGs can also inhibit the growth of tumor cells without US, the tumor inhibition effect was quite limited. The US‐mediated antitumor effect was also observed by live–dead staining (Figure 4F). The experimental MHN + US group showed higher tumor death level compared with the former three groups.

### 2.5 Cellular NO Production, Tumor Pyroptosis, and cGAS‐STING Activation

**Figure 5:**
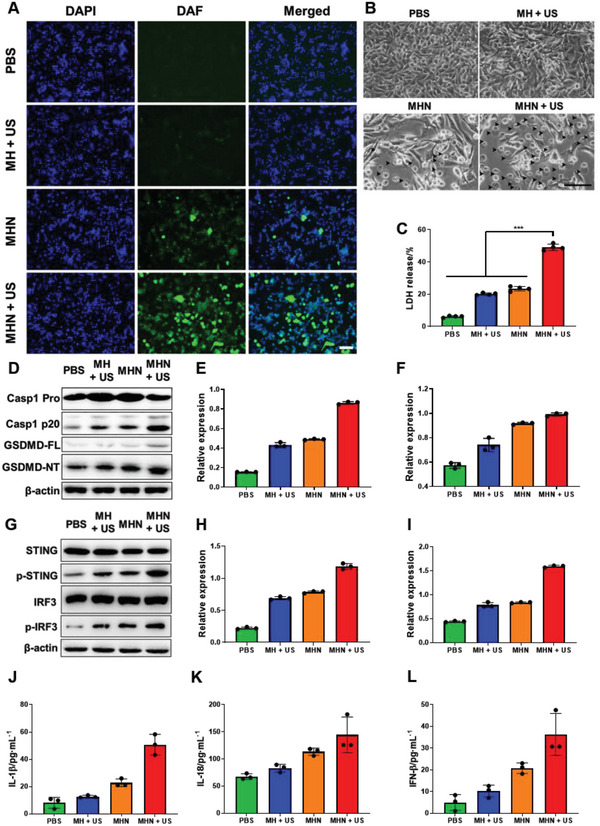
Cellular NO production, tumor pyroptosis, and cGAS‐STING activation of MHN NGs. B16F10 cell were cultured with indicated NGs (40 µg mL^−1^) accompanied by US irradiation. A) Cellular NO production was detected by DAF‐FM DA fluorescence probe. B) Bright filed images of pyroptosis cells incubated with different treatments (black arrows: puffy cells with bubbles protruding from plasm membrane, 200×, scale bar: 100 µm). C) LDH release from pyroptosis cells with different treatment quantitated by LDH cytotoxicity assay kit (*n* = 4). D) Western blot analysis of pyroptosis‐related proteins, including caspase‐1, caspase‐1 p20 (cleaved caspase‐1), GSDMD, GSDMD‐NT (cleaved GSDMD). Quantitative analysis of E) caspase‐1 p20 and F) GSDMD‐NT expression normalized to *β*‐actin (*n* = 3). G) Western blot analysis of cGAS‐STING‐related proteins, including STING, phosphorylation of STING (p‐STING), IRF3, and IRF3, and phosphorylation of IRF3 (p‐IRF3). Quantitative analysis of H) p‐STING and I) p‐IRF3 expression normalized to *β*‐actin (*n* = 3). Enzyme‐linked immunosorbent assay (ELISA) cytokines assay for J) IL‐1*β*, K) IL18, and L) IFN‐*β* of B16F10 cells with US treatment (*n* = 3).

Cellular NO production of MHN NGs in tumor cells was detected by fluorescent staining and NO assay kit. Diaminofluorescein‐FM diacetate (DAF‐FM DA) was used as a NO fluorescent staining probe to monitor the cellular NO amount. Compared with the US‐treated MH group, MHN with US irradiation showed a sharp increase in fluorescent intensity (**Figure** **5**A), proving the efficient cellular NO production by MHN NGs. The bare MHN group showed a modest increase in fluorescent intensity compared with the US‐treated MHN group, indicating that the NO production by MHN NGs was efficiently activated by US. The NO production was also be quantified by NO assay kit, showing the same trend aforementioned (Figure S10, Supporting Information).

Excess NO production may cause local cellular nitric stress to induce pyroptosis.^[^ ^36^ ^]^ The cell morphology was observed by optical microscope with different treatments 18 h after US irradiation (Figure 5B). Time‐dependent pyroptosis was also measured (Figure S11A–F, Supporting Information). The arrows indicated the swollen cells with bubbles protruding from plasm membrane, which is the typical symbol of pyroptosis cells. The experimental MHN + US group showed a higher percentage of puffy cells. The time‐dependent pyroptosis property of MHN NGs was then quantified by western blot assays (Figure S12, Supporting Information). After incubation for 12 h, the GSDMD‐cleaved protein was significantly increased after MHN + US treatment indicating the pyroptosis, which is consistent with the morphology observation results in Figure S11 in the Supporting Information. Lactate dehydrogenase (LDH) is a typical cytoplasm enzyme which can be released to extracellular matrix by the pyroptosis cells.^[^ ^37^ ^]^ Thus, we used LDH cytotoxicity assay kit to quantitatively measure the LDH level in the supernatant, which also revealed the pyroptosis level (Figure 5C). Combined with US, MHN NGs showed a significant pyroptosis effect toward tumor cells compared with other groups.

Caspase‐1 is activated during the pyroptosis process to cause the cleavage of gasdermin (GSDM) family proteins and the synthesis of inflammatory cytokines.^[^ ^38^ ^]^ The expression of Cleaved Caspase‐1 (casp1 p20) and N‐terminal of GSDMD (GSDMD‐NT) was detected by western blot to further verify the function of MHN with US irradiation. The expression of casp1 p20 and GSDMD‐NT of the US‐treated MHN group significantly increased compared with the bare MHN‐treated group (Figure 5D–F), which was attributed to the sufficient production of NO. Meanwhile, the production of inflammatory cytokines, including interleukin‐1*β* (IL‐1*β*) and IL‐18 also increased under the treatment of MHN + US (Figure 5J,K).

cGAS‐STING is an intrinsic signaling pathway detecting the dsDNA and activating the host immunity by secretion of multiple immunomodulation factors, especially the type I interferon (IFN), which can modulate the immune activity for antitumor therapy.^[^ ^39^ ^]^ The expression of phosphorylated STING (p‐STING) and downstream interferon regulating factor 3 (p‐IRF3) suggest the activeness of cGAS‐STING pathway.^[^ ^40^ ^]^ As shown in Figure 5G, the US‐treated MH group upregulated the expression of p‐STING and p‐IRF3, which is mainly caused by the released Mn^2+^ ions as an activator of cGAS.^[^ ^41^ ^]^ The US‐treated MHN group upregulated the expression of p‐STING (Figure 5H and Figure S12, Supporting Information) and p‐IRF3 (Figure 5I) compared with the US‐treated MH group. It may be due to released dsDNA from pyroptosis tumor cells under US irradiation. The protein of p‐STING increased after 12 h, and peak of effective cGAS‐STING activation seems to be delayed compared with the pyroptosis approach of B16F10 cells (Figure S12, Supporting Information). These data demonstrate the time order of pyroptosis and cGAS‐STING immunotherapy, which is an evidence for the proposed cascade therapeutic method. MHN NGs can not only act as the Mn^2+^ ion donors, but also produce NO under US irradiation to provide dsDNA. The synergistic function of Mn^2+^ ions and NO‐triggered pyroptosis contributed to the activation of STING and the subsequent production of IFN‐*β* (Figure 5L). The results above indicated that MHN NGs with US treatment had the ability to achieve cascade pyroptosis and cGAS‐STING tumor therapy.

### 2.6 In Vivo Biodistribution and Antitumor Studies

**Figure 6:**
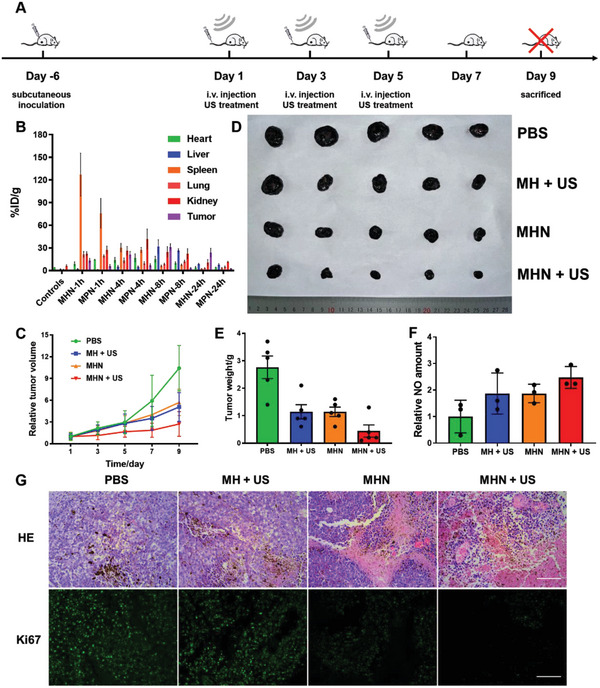
Tumor growth inhibition of MHN NGs with US irradiation in vivo. A) Schematic illustration of in vivo tumor therapeutic effect of MHN NGs for B16F10‐bearing mice under US treatment for 9 days (*n* = 5). B) In vivo biodistribution and pharmacokinetics studies of MHN NGs detected by ICP‐MS with Mn element %ID g^−1^ in major organs (heart, liver, spleen, lung, and kidney) and tumors (*n* = 5). C) Tumor volume changing curve of tumor‐bearing mice during 9 days (*n* = 5). Mice D) tumor morphology images and E) weight measurement (*n* = 5) at 9th day after tumor extraction and F) relative NO amount in tumors after US irradiation (*n* = 5). G) Representative HE staining and Ki‐67 immunofluorescence staining of tumor samples, scale bar: 100 µm.

The B16F10 cells were subcutaneously injected to establish tumor‐bearing mice. The mice with 80–100 mm^3^ tumor were used for further experiments. Based on the good tumor targeting ability in vitro, biodistribution of MHN NGs in vivo was established. The tumor‐bearing mice were randomly divided into two groups and intravenously injected with MPN NGs and MHN NGs. Mice were sacrificed 1, 4, 8, and 24 h after injection with major organs (heart, liver, spleen, lung, and kidney) and tumors extracted to monitor the in vivo biodistribution. The Mn amount in extracted organs and tumors were measured by inductively coupled plasma‐mass spectrometry (ICP‐MS) after cytolysis (Figure S13, Supporting Information). The percentage of injected dose per gram (%ID g^−1^) was then evaluated (**Figure** **6**B). The distribution of MPN and MHN NGs in major organs seemed to be similar, but the dose of MHN NGs in tumor at 8 h was 23.85 ± 3.70% g^−1^, eight times higher than that of MPN NGs. This result confirmed the active targeting ability of HA, and 8 h was chosen as the US treatment time period after injection. Moreover, we discovered the low‐level dose of MPN and MHN NGs in major organs after 24 h circulation, which revealed that both NGs were able to be cleared from major organs. This may be due to the degradation effect of hMnO_2_ NPs that can reduce the side effects in body circulation after injection.

Encouraged by the tumor cell inhibition results in vitro, tumor inhibition property of MHN NGs was further evaluated in vivo. The tumor‐bearing mice were randomly divided into four groups (*n* = 5). The prepared NGs were intravenously injected every 2 days. After 8 h of injection, US treatment was applied for indicated groups (Figure 6A). When tumor volumes reached ethical limits at the 9th day, mice in each group were sacrificed, and the tumor volumes during the treatment process were monitored (Figure 6C). The tumor image (Figure 6D) and weight (Figure 6E) on the 9th day were detected. The MHN + US group showed the minimum size of tumors compared with other groups, suggesting its great tumor inhibition property. In addition, the intracellular NO amount in tumor samples was detected (Figure 6F). The NO concentration in MHN + US group was augmented compared with the control group, which further proved the efficient NO production of MHN with US irradiation.

Besides, hematoxylin and eosin (HE) staining and Ki67 immunofluorescence staining were applied to show the histomorphological change of tumor tissues. HE staining showed the most severe tumor tissue structural damage and necrosis treated with MHN NGs under US irradiation (Figure 6G). The US‐treated MHN group also showed the weakest Ki‐67 immunofluorescence intensity, suggesting great tumor inhibition efficiency (Figure 6G).

To evaluate the biosafety of MH NGs and MHN NGs, a series of experiments was conducted. The weight of mice in NGs‐treated groups kept similar to that in the control groups (Figure S14, Supporting Information). The blood routine examinations and blood biochemical examinations were conducted. The number of white blood cells (WBC), red blood cells (RBC), and platelet (PLT) of both NGs kept similar to that of control group, indicating great compatibility of MHN NGs (Figure S15A–C, Supporting Information). The indexes of alanine aminotransferase (ALT), aspartate aminotransferase (AST), creatinine (CRE), and blood urea of MH NGs and MHN NGs treated mice were close to that of the control group, which showed that NGs did no severe harm to major organs (Figure S16A–D, Supporting Information). The morphology of major organs remained normal after indicated treatments, which indicated good biocompatibility of this therapeutic nanoplatform (Figure S17, Supporting Information).

### 2.7 In Vivo Pyroptosis and STING Activation

**Figure 7:**
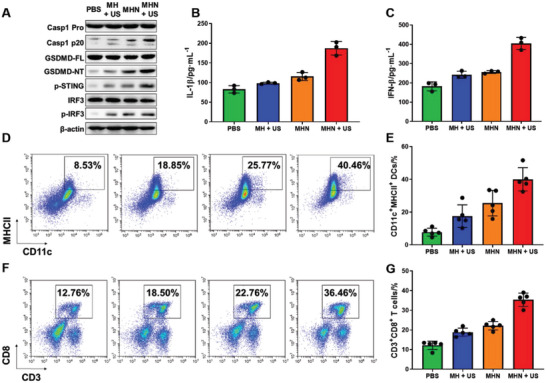
Pyroptosis and STING activation of MHN NGs with US irradiation in vivo. A) Western blot assay for GSDMD‐NT, p‐STING, and p‐IRF3 protein expression in tumor tissues with indicated treatments. ELISA cytokines assay of B) IL‐1*β* and C) IFN‐*β* in tumor tissues (*n* = 3). Flow cytometry analysis of D) CD45^+^CD11c^+^MHNII^+^ DCs and F) CD45^+^CD3^+^CD8^+^ T cells in tumor tissues and E,G) statistical analysis (*n* = 5).

To estimate the pyroptosis level in vivo, the expression of pyroptosis‐related proteins in tumor samples was detected. The expression of Caspase‐1 p20 and GSDMD‐NT was significantly upregulated in the US‐treated MHN group compared with the US‐treated MH group (**Figure** **7**A and Figure S18, Supporting Information). It was mainly due to the NO produced by MHN NGs. Simultaneously, the expression of proinflammation cytokines IL‐1*β* upregulated in tumor tissues (Figure 7B). Afterward, the cGAS‐STING pathway was activated with upregulated expression of p‐STING and p‐IRF3 (Figure 7A and Figure S18A–D, Supporting Information). Subsequently, the transcription and expression of IFN‐*β* was increased (Figure 7C). IFN‐*β* is an immunostimulatory cytokine participating in anticancer immunity, which can promote the activation and maturation of dendritic cell (DC), and the infiltration of T cells and acquisition of cytotoxic T lymphocytes (CTLs).^[^ ^42^ ^]^ The activated DC cells (Figure 7D,E) and cytotoxic T cells (Figure 7F,G) in tumor tissues were detected by flow cytometry. There were more activated DCs and CTLs in tumor tissues treated with MH + US. It was mainly due to the released Mn^2+^ ions from MH. And the proportion of DCs and CTLs was further improved with the treatment of MHN + US, which revealed the synergetic immunotherapy property of this nanoplatform. As a result, the in vivo cascade pyroptosis and STING activation effect of MHN NGs was certified.

## 3 Conclusion

In summary, the US‐sensitive NO donor NMA with the designed structure has been selected and loaded into HA‐modified hMnO_2_ NPs to fabricate MHN NGs. The obtained NGs have a record‐high NO generation property under US irradiation among different US‐triggered NO donors. The MHN NGs can target tumor sites and release NMA and Mn^2+^ quickly in the TME. The in vitro and in vivo studies demonstrate that the MHN NGs can activate the cascade tumor pyroptosis and cGAS‐STING immunotherapy under the US treatment through the synergistic effects of generated NO and released Mn^2+^, resulting in the effective inhibition of tumor growth. This work offers a molecular simulation method for US‐triggered NO donor selection, and gives guidance to noninvasive NO precise delivery and NO‐based therapeutic nanoplatforms for combined cancer therapy.

## 4 Experimental Section

### Synthesis of sSiO_2_ NPs

sSiO_2_ NPs were synthesized as referred to previous studies.^[^ ^34d^ ^]^ Typically, 0.5 mL tetraethyl orthosilicate was added to the mixture of 100 mL ethanol and 5 mL deionized water in a 250 mL flask, and the solution was stirred for 0.5 h. Then, 5 mL NH_3_·H_2_O was added dropwise into above solution and the flask was kept stirring for another 1 h at 25 °C. The product was obtained by centrifugation at 10 000 rpm for 10 min and washed by ethanol for three times.

### Synthesis of hMnO_2_ NPs

To obtain MnO_2_ shell, 40 mg sSiO_2_ NPs were dispersed in 80 mL deionized water and stirred for 0.5 h in a 250 mL flask. Then, 10 mL 30 mg mL^−1^ KMnO_4_ aqueous solution was added dropwise in the above solution and the flask maintained stirring for 6 h at 25 °C. The obtained dispersion was centrifuged and washed by deionized water for three times to remove the remaining reactants. Later, the core–shell sSiO_2_@MnO_2_ product was dispersed in 100 mL 0.2 m NaOH for 12 h at 25 °C to etch the SiO_2_ template, and hMnO_2_ NPs were obtained by washing the dispersion three times by deionized water.

### Synthesis of hMnO_2_@HA (MH) Nanoplatforms (NGs)

PAH was used for surface modification first. Typically, 20 mg hMnO_2_ NPs were added to 40 mL 0.25 mg mL^−1^ PAH solution, and the reaction was kept for 1 h at 25 °C under intense stirring. The product was washed by deionized water for three times. Later, 10 mg HA was dissolved in 40 mL deionized water, and then 4.8 mg 1‐ethyl‐3‐(3‐dimethylaminopropyl)carbodiimide and 5.4 mg *N*‐hydroxysuccinimide were added to stir at 25 °C for 0.5 h. The aforementioned product was added to this solution and reacted for 12 h at 25 °C. Finally, MH NGs were obtained by centrifugation and washed by deionized water for three times. MP NGs were synthesized with the same procedure except using PAA instead of HA.

### Loading of NMA

First, 4 mg MH NGs were dispersed in 16 mL deionized water, and moderate amount of NMA in 0.2 mL ethanol was added dropwise to the solution. After stirring at 25 °C for 24 h, the MH@NMA (named MHN NGs were centrifuged and washed by deionized water for three times. MP@NMA (named MPN) NGs were fabricated with the same procedure except using MP NGs instead of MH NGs.

### Degradation, Fenton, and Catalase‐Like Activity Studies

Degradation activity of MHN NGs was measured by UV‐Vis spectrum. Meanwhile, 0.5 mg MHN NGs were dissolved in 10 mL TME‐mimicking buffer (PBS, pH = 5.0, with 100 × 10^−6^ m H_2_O_2_) and neutral buffer (PBS, pH = 7.0), respectively. The absorption at 360 nm was monitored by UV‐Vis spectrophotometer for 240 s. The experiment was repeated for three times.

Fenton‐like activity of MHN NGs was measured by methylene blue (MB) degradation. Different concentrations of MHN NGs (0, 50, 100, 200 mg mL^−1^) were added to TME‐mimicking buffer (PBS, pH = 5.0, with 200 × 10^−6^ m H_2_O_2_) with 0.1 × 10^−3^ m MB, and the absorption at 664 nm was detected. Then, different concentrations of H_2_O_2_ (0 × 10^−3^, 100 × 10^−3^, 200 × 10^−3^, 400 × 10^−3^ m) were added to TME‐mimicking buffer (PBS, pH = 5.0) with 0.1 × 10^−3^ m MB and 200 mg mL^−1^ MHN NGs, and the absorption at 664 nm was detected.

Catalase‐like activity of MHN NGs was analyzed by dissolved oxygen analysis. For experiment group, 0.5 mg MHN NGs were added into 10 mL TME‐mimicking buffer (PBS, pH = 5.0, with 100 × 10^−6^ m H_2_O_2_). The dissolved oxygen was measured by dissolved oxygen meter every 30 s, and the total measurement time was 6 min. To compared, mere buffer solution was detected as the control group.

### NMA Release and NO Production Studies

Typically, 10 mg MHN NGs were dissolved in 1 mL TME‐mimicking buffer (PBS, pH = 5.0, with 100 × 10^−6^ m H_2_O_2_) and then added to a dialysis bag. The dialysis bag was then immersed in another 19 mL buffer and incubated at 37 °C with constant vibration. At 0, 1, 2, 4, 8, and 24 h, 1 mL leaching solution was extracted and the equivalent buffer was added. The leaching solution was detected by UV‐Vis spectrophotometer to quantify the released NMA amount. The experiment was repeated for three times.

NO production was detected with NO assay kit. Briefly, 100 µL 0.5 mg mL^−1^ MHN NGs and equivalent PBS were, respectively, added to 96‐well plate. After adding equivalent NO assay kit, half of each group was then treated with planar US (*I* = 1 W cm^−2^, *S* = 5 cm^2^, 3 MHz) for 5 min. UV‐vis absorption at 540 nm was measured immediately by a microplate reader. The experiment was repeated five times. The NO production with different US irradiation times and intensities was also evaluated.

### In Vitro Cytotoxicity Studies

Cytotoxicity studies were conducted by 3T3 cells and B16F10 cells. Tested cells were, respectively, cultured in 96‐well microplates with 10^4^ cells per well. Then, different concentrations of MHN NGs (5, 10, 20, 40, 80, and 160 µg mL^−1^) and 1 × PBS were added to each well and each group was incubated at 37 °C under 5% CO_2_. After 24 h, MHN NGs were washed by PBS and 10 µL CCK‐8 reagent was added to the plate and further incubated for 1 h. To measure the viability of cells, the absorbance at 450 nm was determined by microplate reader.

### Tumor Cellular Uptake and Antitumor Studies

Tumor cellular studies were conducted by using B16F10 cells and 3T3 cells. For tumor cellular uptake studies, B16F10 cells and 3T3 cells were cultured in 12‐well microplates and, respectively, added by MHN NGs and MPN NGs (40 µg mL^−1^) dyed with equivalent Nile Red. After co‐incubated for 1, 3, and 6 h, NPs were washed by PBS and 4′,6‐diamidino‐2‐phenylindole dyes were then added to stain the cell nucleus. The remaining dyes were washed after incubation for 30 min and confocal microscope images were obtained. The intracellular fluorescence intensity was analyzed by flow cytometry.

Intracellular reactive ROS were detected in B16F10 cells. B16F10 cells were cultured in 12‐well plates with 5 × 10^5^ cells per well. Then, different concentrations of MHN NGs (5, 10, 20, 40, 80, and 160 µg mL^−1^) and 1 × PBS were added to each well and incubated at 37 °C under 5% CO_2_. After 12 h, MHN NGs were washed with PBS and dichlorodihydrofluorescein diacetate probe (S0033, Beyotime) was added to the plate and further incubated for 30 min. Afterward, cells were collected and analyzed by flow cytometry.

For antitumor studies, B16F10 cells were cultured in 96‐well microplates with 10^4^ cells per well. PBS, MH NGs, MHN NGs, and MHN NGs (40 µg mL^−1^) were, respectively, added to each well. After incubation for 12 h, the 2nd and 4th groups were exposed with planar US (*I* = 2.5 W cm^−2^, *S* = 1 cm^2^, 3 MHz) for 5 min. After 24 h, the remaining NGs were removed and 10 µL CCK‐8 reagent was added to each group. After co‐incubated for 1 h, the absorbance at 450 nm was measured by a microplate reader. The live–dead staining of each group was studied by the same treatment method except for using 12‐well cell plate. After 24 h, the remaining NGs were removed and calcein AM/PI double stain kit was added to each group. The images were then captured by a confocal microscope.

### Cellular NO Detection

DAF‐FM DA fluorometric assay kit was used for cellular NO fluorescent staining. B16F10 cells were cultured in 12‐well microplates, and then PBS, MH NGs, MHN NGs, and MHN NGs (40 µg mL^−1^) were, respectively, added to each well. The 2nd and 4th group were exposed with planar US (*I* = 2.5 W cm^−2^, *S* = 1 cm^2^, 3 MHz) for 5 min. After 24 h, the remaining NGs were removed and DAF‐FM DA probe was added to each group. The confocal microscope images were then obtained by inverted fluorescent microscopy.

Cellular NO production was also measured quantitatively with NO assay kit. B16F10 cells were cultured in 96‐well microplates with 10^4^ cells per well. PBS, MH NGs, MHN NGs, and MHN NGs (40 µg mL^−1^) were, respectively, added to each well and the 2nd and 4th groups were exposed with planar US (*I* = 2.5 W cm^−2^, *S* = 1 cm^2^, 3 MHz) for 5 min. After 24 h, the remaining NGs were removed and the cells were collected and lysed. The NO assay kit was then added to each group. After co‐inhibited for 30 min, the absorbance at 540 nm was measured by a microplate reader to quantify cellular NO.

### Cellular Pyroptosis and cGAS‐STING Activation

Morphology of B16F10 cells was observed by optical microscope. B16F10 cells were cultured in 12‐well microplates, and then PBS, MH NGs, MHN NGs, and MHN NGs (40 µg mL^−1^) were, respectively, added to each well. The 2nd and 4th group were exposed with planar US (*I* = 2.5 W cm^−2^, *S* = 1 cm^2^, 3 MHz) for 5 min. After 24 h, the morphology of B16F10 cells of each group was captured. The time‐dependent morphology of the 4th group was monitored at 2, 4, 8, 12, 18, and 24 h to detect the cellular pyroptosis. The protein expression of caspase‐1, caspase‐1 p20, GSDMD, GSDMD‐NT, *β*‐actin, STING, p‐STING, IRF3, and p‐IRF3 in B16F10 cells were detected by western blot. Briefly, the collected cells were lysed by radioimmunoprecipitation assay lysis buffer and subjected to the sodium dodecyl sulfate‐polyacrylamide gel electrophoresis and converted to the polyvinylidene fluoride film. The indicated antibodies caspase‐1 p20 (22915‐1‐AP, Proteintech), GSDMD‐NT (HA‐721144, Huabio), STING (13647S, CST), p‐STING (72971S, CST), IRF3 (ET1612‐14, Huabio), p‐IRF3 (4947, CST), and *β*‐actin (GB12001, Servicebio) were applied and incubated at 4 °C overnight. The IL‐1*β*, IL‐18, and IFN‐*β* were detected via the murine IL‐1*β* (88‐7013, Invitrogen), IL‐18 (EK218, Multi Sciences), and IFN‐*β* (EK2236, Multi Sciences) ELISA kit, respectively, according to the manufacturer's instructions.

### Animal Model Establishment

All animal experiments in this work were processed according to the guidelines approved by the ethics committee of laboratory animals, the Second Affiliated Hospital of Zhejiang University School of Medicine. For the establishment of tumor model, the C57BL mice (female, 20 ± 2 g, 3–4 weeks) were subcutaneously inoculated by 10^6^ B16F10 cells at the axilla of mice. The modeled mice were used for further experiments after about 7 days when the tumor volume reached 80–100 mm^3^.

### In Vivo Biodistribution and Biosafety Studies

After tumor models were established, mice were averagely dispersed in nine groups (*n* = 5). One group of mice was sacrificed at the beginning as the control group. Later, the dose of 10 mg kg^−1^ MHN NGs and MPN NGs were injected by tail vein to the remaining four groups of the tumor‐bearing mice, respectively. The biodistribution and pharmacokinetic studies were obtained by ICP‐MS. At 1, 4, 8, and 24 h after injection, one group of both MHN NGs and MPN NGs was sacrificed and the major organs and tumors were obtained. The Mn amount in each organ and tumor was measured by ICP‐MS, and the %ID g^−1^ value was then calculated. After intravenous injection of MH NGs and MHN NGs for three times, blood of mice was obtained for biochemical examinations and blood routine examinations to evaluate the in vivo biosafety property of both NGs. The major organs (including heart, liver, spleen, lung, kidney) were also extracted for HE staining.

### In Vivo Tumor Therapy Study

The tumor‐bearing model mice were randomly divided into four groups (*n* = 5): A) 1 × PBS; B) MH NGs (10 mg kg^−1^) + US; C) MHN NGs (10 mg kg^−1^); D) MHN NGs (10 mg kg^−1^) + US. The prepared NGs were injected by tail vein every 2 days to each group for three times. After 8 h of injection, US treatment (*I* = 2.5 W cm^−2^, *S* = 1 cm^2^, 3 MHz, 5 min) was applied on groups B and D. Tumor volume and mice weight were measured every 2 days before intravenous injection. After 9 days, mice in each group were sacrificed and solid tumors were used for HE staining and Ki67 immunofluorescence staining and flow cytometry study. NO amount of each group was also measured by NO assay kit after tumor cytolysis. Solid tumors after last US irradiation were used for NO amount analysis by NO assay kit after tumor cytolysis. The activated DCs and CTLs in tumor were detected. Briefly, extracted tumors were lysed to single cell and incubated with indicated flow cytometry antibodies, including CD45 (564225, BD Pharmingen), CD11c (557401), MHC‐II (562564), CD3 (553061), CD8 (551162) for flow cytometry analysis.

### Statistical Analysis

Analyses were performed with GraphPad Prism 8. Sample size (*n*) for each statistical analysis was no less than 3. Sample data were all presented by mean value ± standard deviation (e.g., mean ± SD). Statistical analysis for comparing two experimental groups was performed using two‐sided student's *t* tests. In experiments with multiple groups, one‐ or two‐way analysis of variance with Tukey multiple comparisons was used to calculate the differences. Differences were labeled as * for *p* < 0.05, ** for *p* < 0.01, *** for *p* < 0.001.

## Conflict of Interest

The authors declare no conflict of interest.

## Supporting information

Supporting Information

## Data Availability

The data that support the findings of this study are available from the corresponding author upon reasonable request.
