# Supplementary material for: Record‐High Ultrasound‐Sensitive NO Nanogenerators for Cascade Tumor Pyroptosis and Immunotherapy
Source: Adv Sci (Weinh). 2023 Jul 3;10(26):2302278. doi: 10.1002/advs.202302278 (PMC10502831; doi:10.1002/advs.202302278)
Supplement: Supplementary file 1 — Supporting Information [file ADVS-10-2302278-s001.pdf]

ADVANCED SCIENCE
Open Access

Supporting Information

for *Adv. Sci.*, DOI 10.1002/advs.202302278

Record-High Ultrasound-Sensitive NO Nanogenerators for Cascade Tumor Pyroptosis and Immunotherapy

Yuheng Bao, Yanni Ge, Mengjie Wu, Zhengwei Mao, Juan Ye* and Weijun Tong**

Supporting Information

Record-high Ultrasound-sensitive NO Nanogenerators for Cascade Tumor Pyroptosis and Immunotherapy

Yuheng Bao[a #], Yanni Ge[b #], Mengjie Wu[c *], Zhengwei Mao[a], Juan Ye[b *], Weijun Tong[a *]

[a] MOE Key Laboratory of Macromolecular Synthesis and Functionalization, Ministry of Education, Department of Polymer Science and Engineering, Zhejiang University, Hangzhou, Zhejiang, China

[b] Eye Center, The Second Affiliated Hospital, School of Medicine, Zhejiang University, Zhejiang Provincial Key Laboratory of Ophthalmology, Zhejiang Provincial Clinical Research Center for Eye Diseases, Zhejiang Provincial Engineering Institute on Eye Diseases, Hangzhou, Zhejiang, China

[c] Stomatology Hospital, School of Stomatology, Zhejiang University School of Medicine, Zhejiang Provincial Clinical Research Center for Oral Diseases, Key Laboratory of Oral Biomedical Research of Zhejiang Province, Cancer Center of Zhejiang University, Hangzhou, Zhejiang, China

#These authors contributed equally to this work.

*Corresponding Authors. Email:

tongwj@zju.edu.cn (W. Tong);

yejuan@zju.edu.cn (J. Ye);

wumengjie@zju.edu.cn (M. Wu)

Contents

Cover 1

Contents 2

Materials and Instruments 3

Instrumental Operation..4

Supporting Figures 5

Materials and Instruments

Materials: Tetraethyl orthosilicate (TEOS) , N-hydroxysuccinimide (NHS), PAH (Mw = 15,000), and ammonia solution were purchased from Aladdin Industrial Co., Ltd. HA (40-100 kDa), 1-ethyl-3-(3-dimethylaminopropyl-carbodiimide hydrochloride (EDC) and NMA were obtained from Shanghai Macklin Biochemical Co., Ltd. PAA (Mw = 450,000) and IR780 iodide were acquired from Sigma Aldrich Co., Ltd. Potassium Permanganate ($KMnO_4$), sodium hydroxide and ethanol were bought from Sinopharm Chemical Reagent Co., Ltd. NO assay kit was purchased from Beyotime Biotech Co., Ltd. Cell counting kit-8 (cck-8) and 4',6-diamidino-2-phenylindole (DAPI) were obtained from ThermoFisher Scientific Co., Ltd. 3-amino,4-aminomethyl-2',7'-difluorescein diacetate (DAF-FM DA) was purchased from Dalian Meilun Biotechnology Co., Ltd. C57BL/6 mice were purchased from Shanghai SLAC Laboratory Animal Co., Ltd. B16F10 cells were obtained from Shanghai institute of biological sciences, Chinese academy of sciences. DMEM cell culture medium and Milli-Q ultrapure water were used throughout the study. All the reagents were used in experiments without further purification.

Instruments: The morphology of NGs was observed by a HT7700 transmission electron microscope (TEM, Hitachi). The chemical structure and element composition were detected by X-ray diffraction (XRD, PANalytical B.V.), UV-Vis spectrophotometer (Hitachi), multifunctional microplate reader (Biotech), Fourier infrared spectrometer (Thermo Electron) and X-ray photoelectron spectroscope (XPS, Thermo Scientific K-Alpha). The size and zeta potential were measured by laser nanometer analyzer (Malvern Panalytical). The pH values of solutions were measured by a FiveEasy pH meter (Mettler Terado). The oxygen production was detected by a Seven2Go pro dissolved oxygen meter (Mettler Terado).

Instrumental Operation

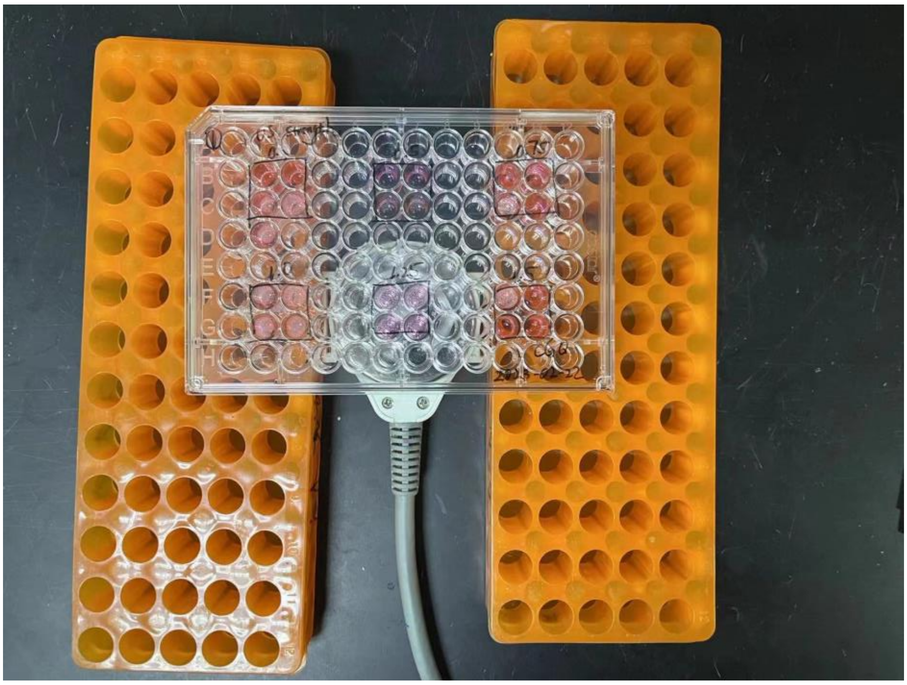

To study the therapeutic effect of MHN NGs under US irradiation, ultrasonic probe was placed upside down on the testing table, and ultrasonic coupling agent was smeared on the top surface. Different microplates were immobilized over the probe (taking 96 well microplate as an example), tightly immersed in the coupling agent. After US procedure was set up, the switch was on and US irradiation treatment was performed.

Supporting Figures

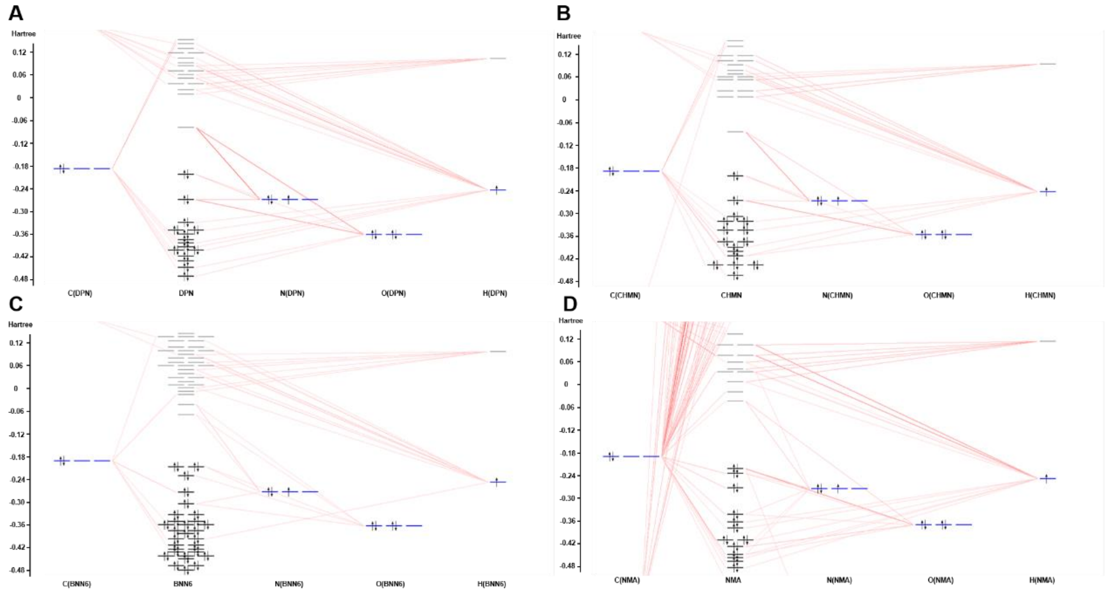

Figure S1 Electron energy level of and band transition energy of DPN (A), CHMN (B), BNN6 (C) and NMA molecules (D) by DFT simulation with AMSjobs.

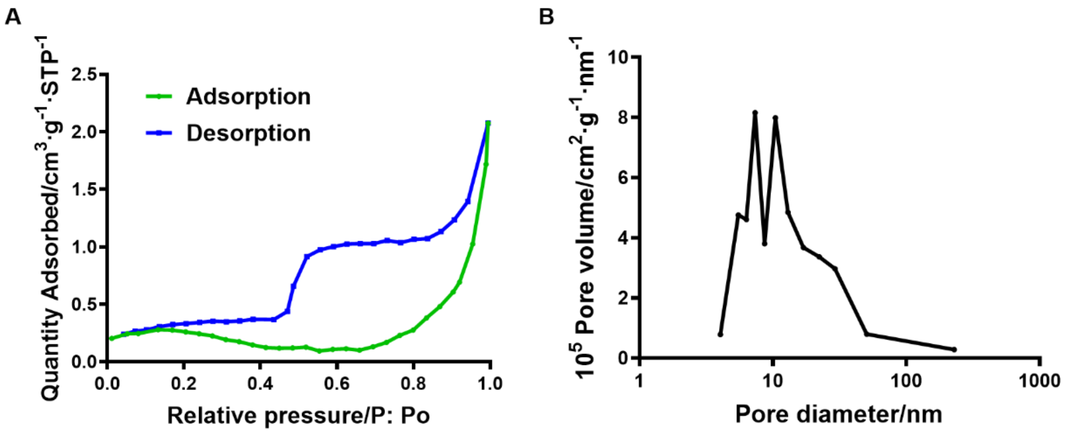

Figure S2 N_2 adsorption/desorption isotherms (inset, A) and pore-size distribution curve (B) of the $hMnO_2$ NPs.

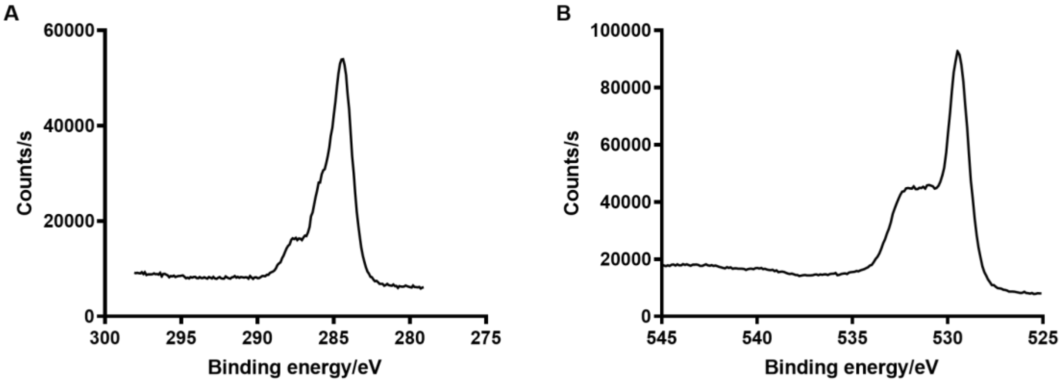

Figure S3 Fine spectra scanning of C1s (A) and O1s (B) of MHN NPs.

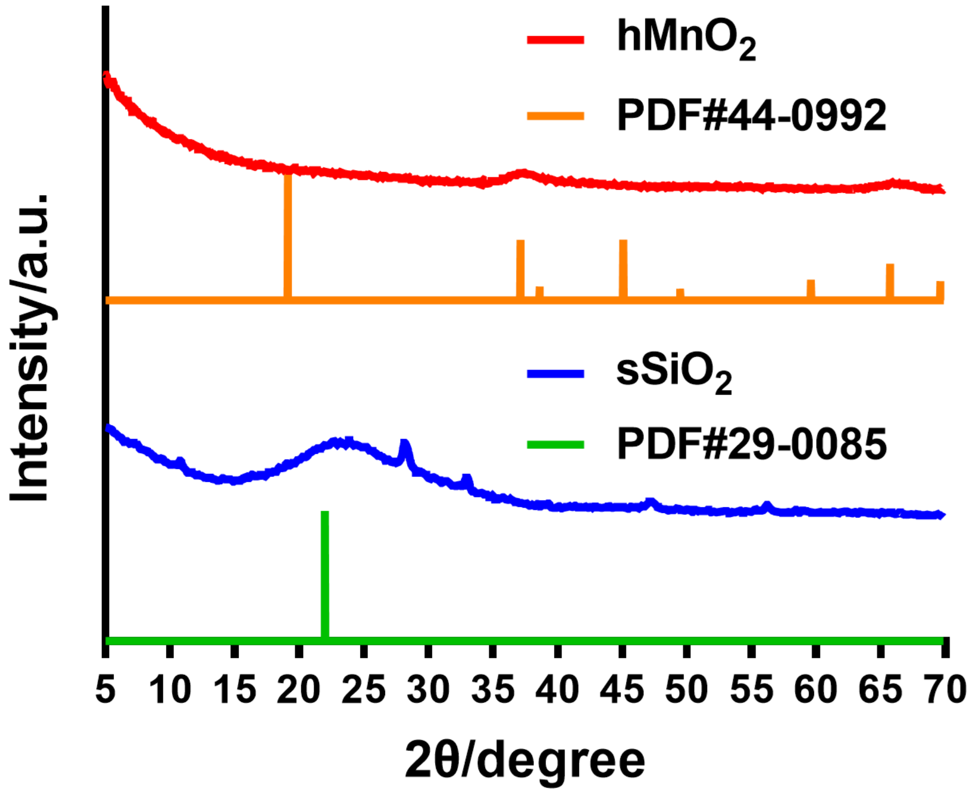

Figure S4 XRD analysis of $sSiO_2$ and $hMnO_2$ compared with standard PDF#29-0085 (SiO_2) and PDF#44-0992 (MnO_2).

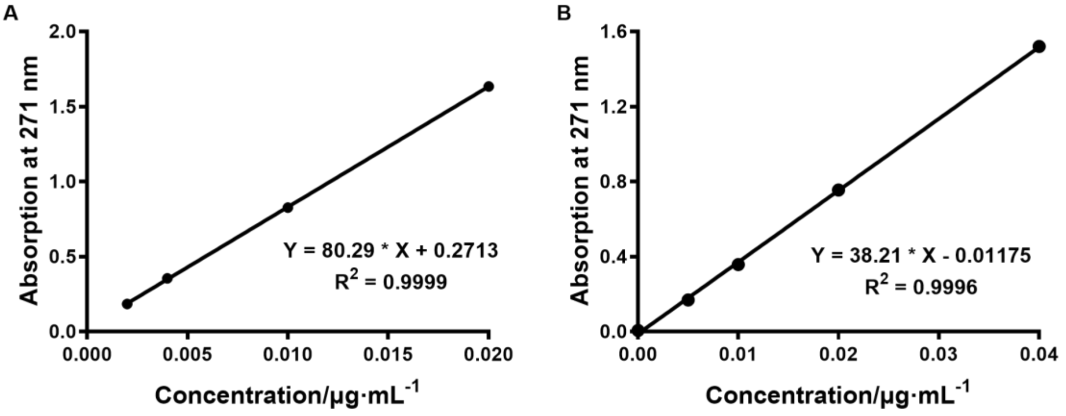

Figure S5 UV-Vis spectrum standard curve of NMA in ethanol (A) and in acid PBS (pH = 5, 100 μM H_2O_2, B).

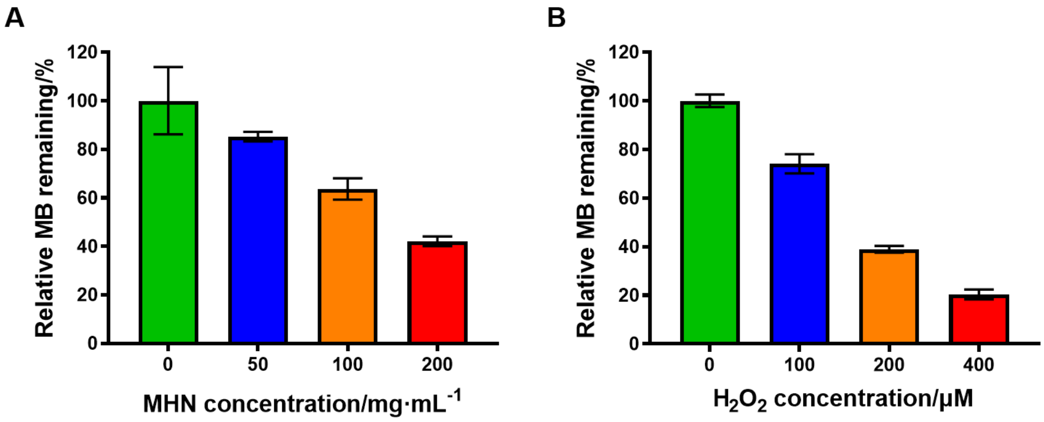

Figure S6 MHN induced ·OH production with different MHN concentration (A) and H_2O_2 concentration (B) by Methylene blue (MB) degradation (n = 3).

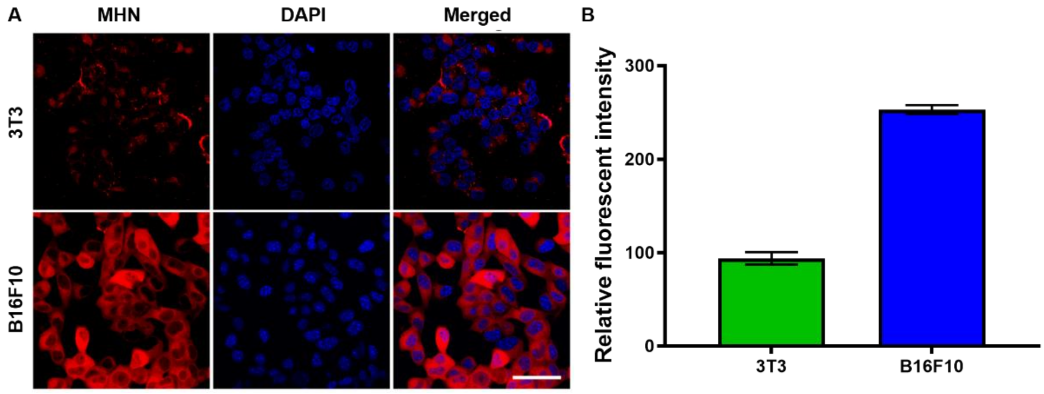

Figure S7 The representative endocytosis images of tumor cells and normal cells after incubation with MHN NGs dyed with Nile Red for 6 h (630 ×, scale bar: 50 μm) (A). Statistical analysis of intracellular fluorescence intensity measured by flow cytometry (n = 3, B).

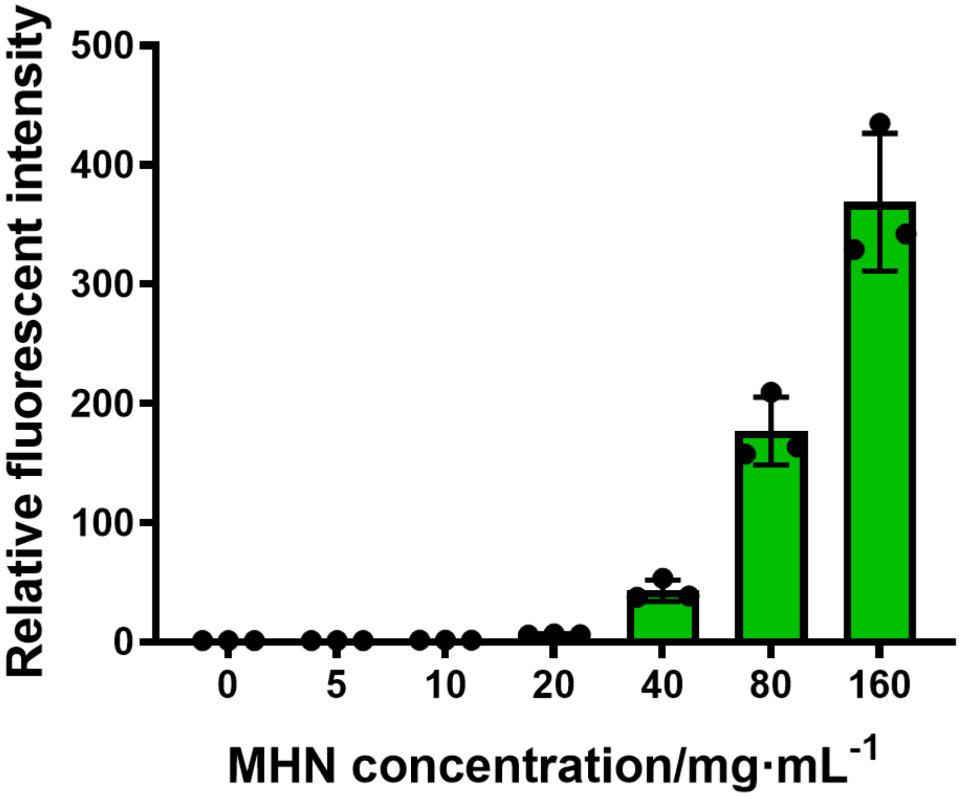

Figure S8 Intracellular reactive oxygen species (ROS) in tumor cells detected by DCFH-DA kit and analyzed by flow cytometry (n = 3).

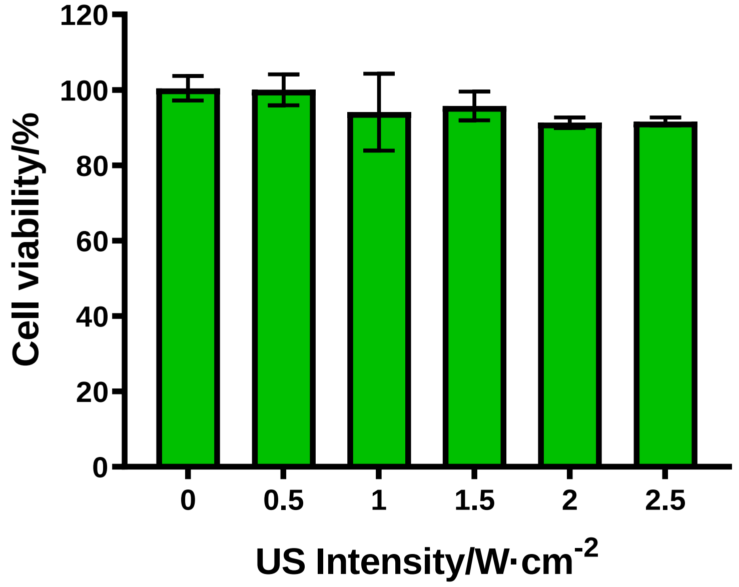

Figure S9 Cellular toxicity of B16F10 cells irradiated by US for different times (n = 5).

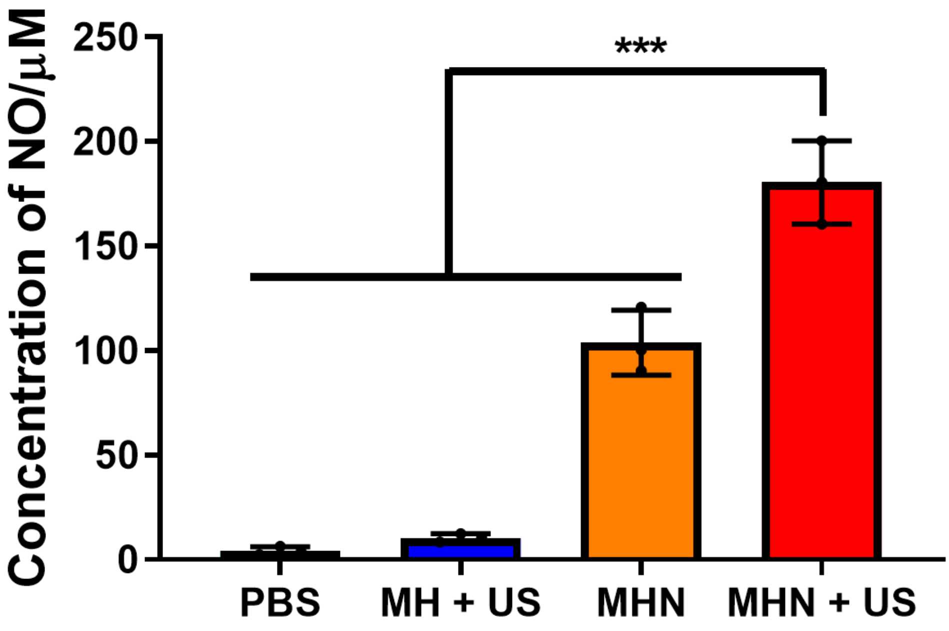

Figure S10 Cellular NO production of MHN NGs combined with US quantitatively measured by NO assay kit (n = 3, ***: $p < 0.001$).

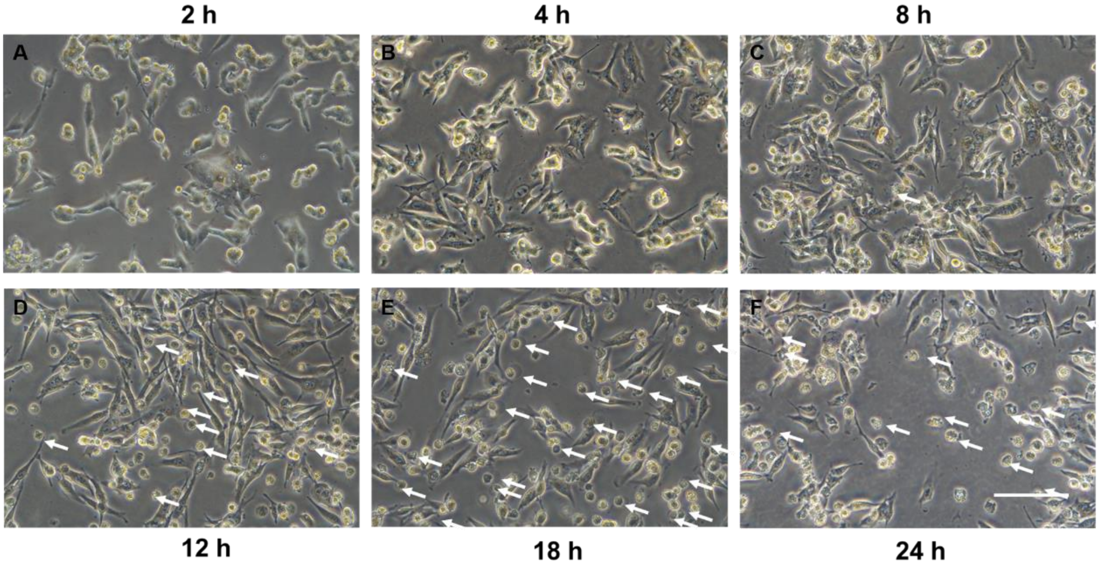

Figure S11 The pyroptosis property of MHN NGs (40 μg/mL) with different treatment times (A: 2 h; B: 4 h; C: 8 h; D: 12 h; E: 18 h; F: 24 h), scale bar: 100 μm.

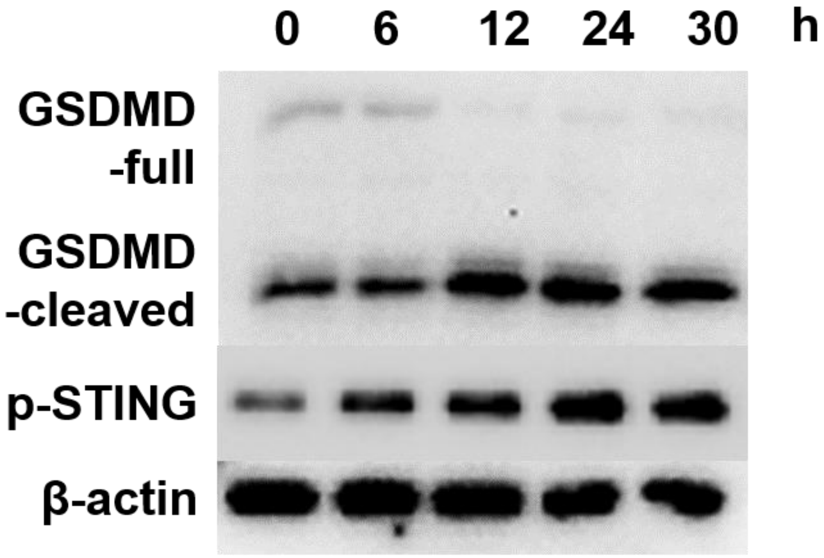

Figure S12 The pyroptosis and cGAS-STING activation properties of MHN NGs (40 μg/mL) with different treatment times quantitatively measured by western blot.

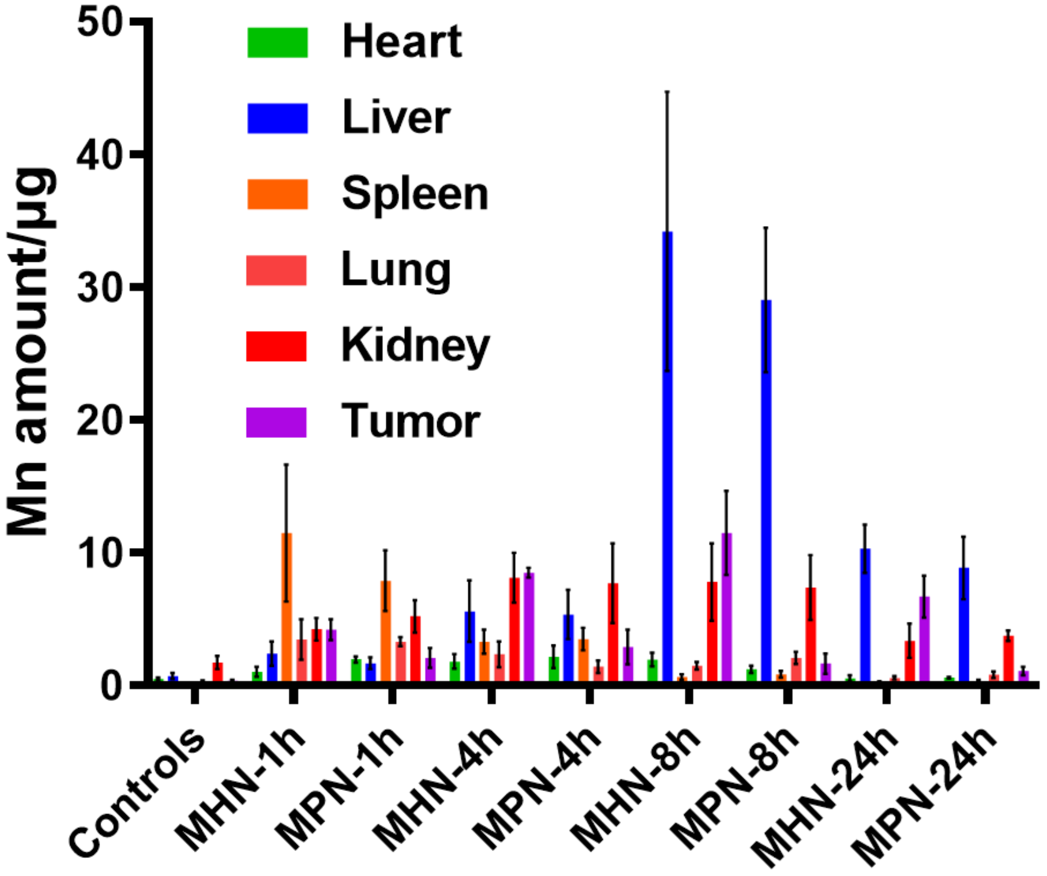

Figure S13 In vivo biodistribution and pharmacokinetics studies of MHN NGs by Mn amount in major organs (heart, liver, spleen, lung, and kidney) and tumors with ICP-MS (n = 5).

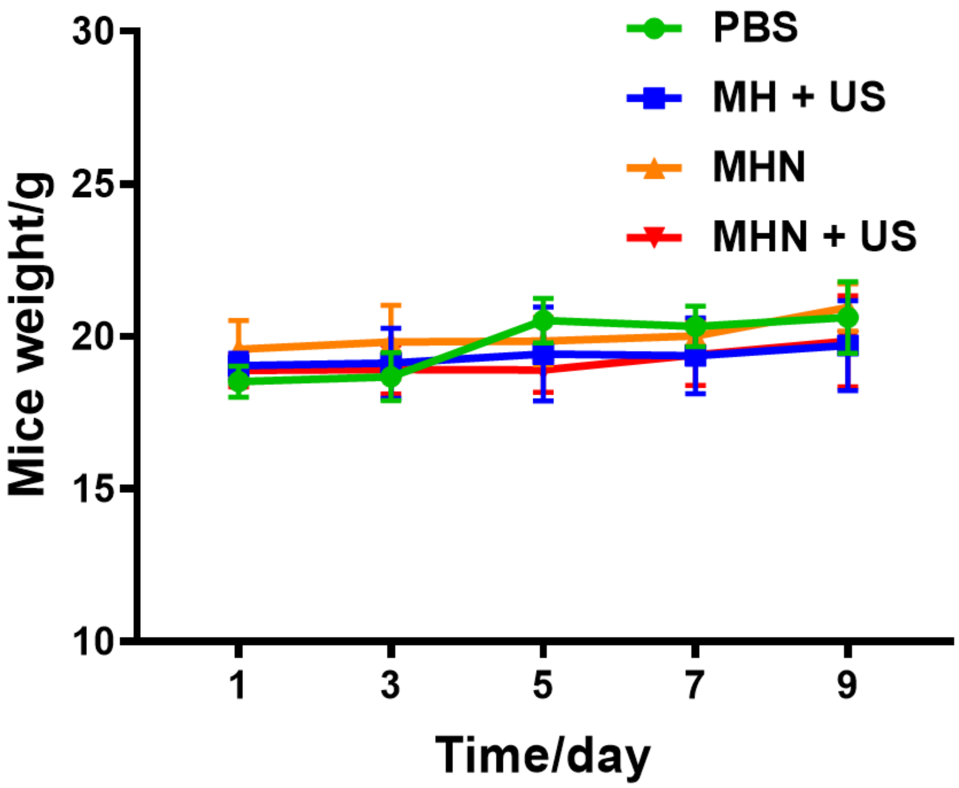

Figure S14 Mice weight changing curve of tumor-bearing mice during 9 days (n = 5).

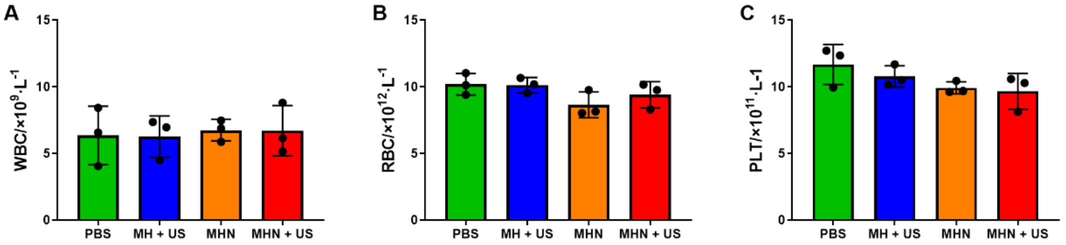

Figure S15 Blood routine examinations of white blood cells (WBC, A), red blood cells (RBC, B) and platelet (PLT, C) (n = 5).

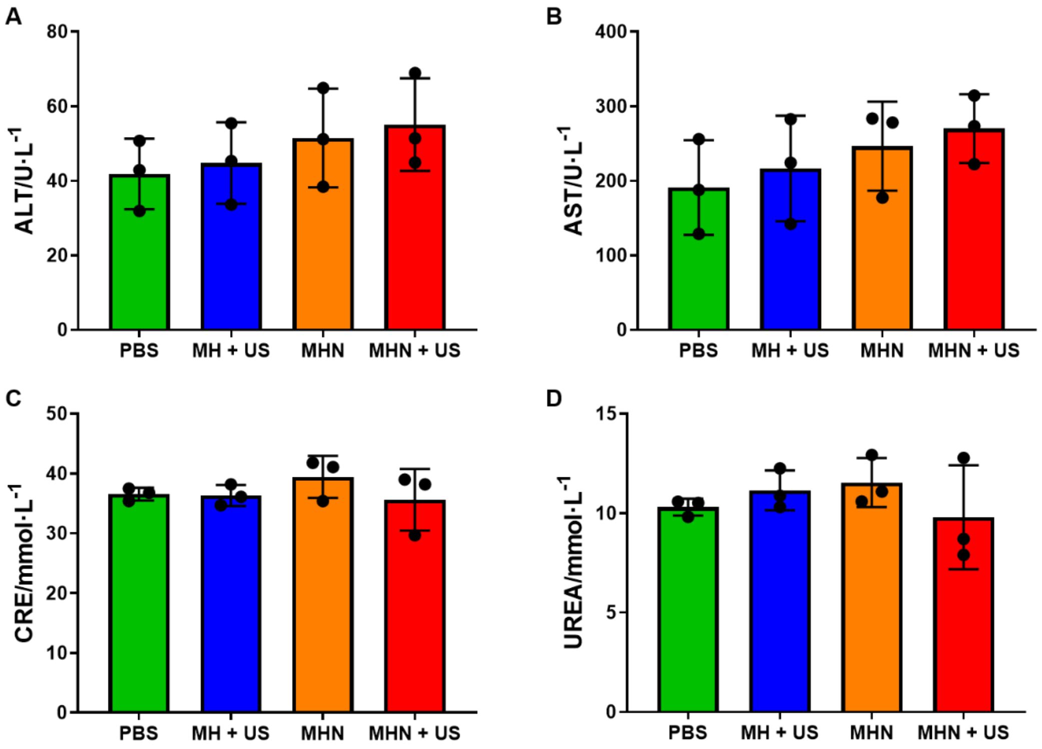

Figure S16 Biochemical examinations of alanine aminotransferase (ALT, A), aspartate aminotransferase (AST, B), creatinine (CRE, C), and blood urea (D) (n = 5).

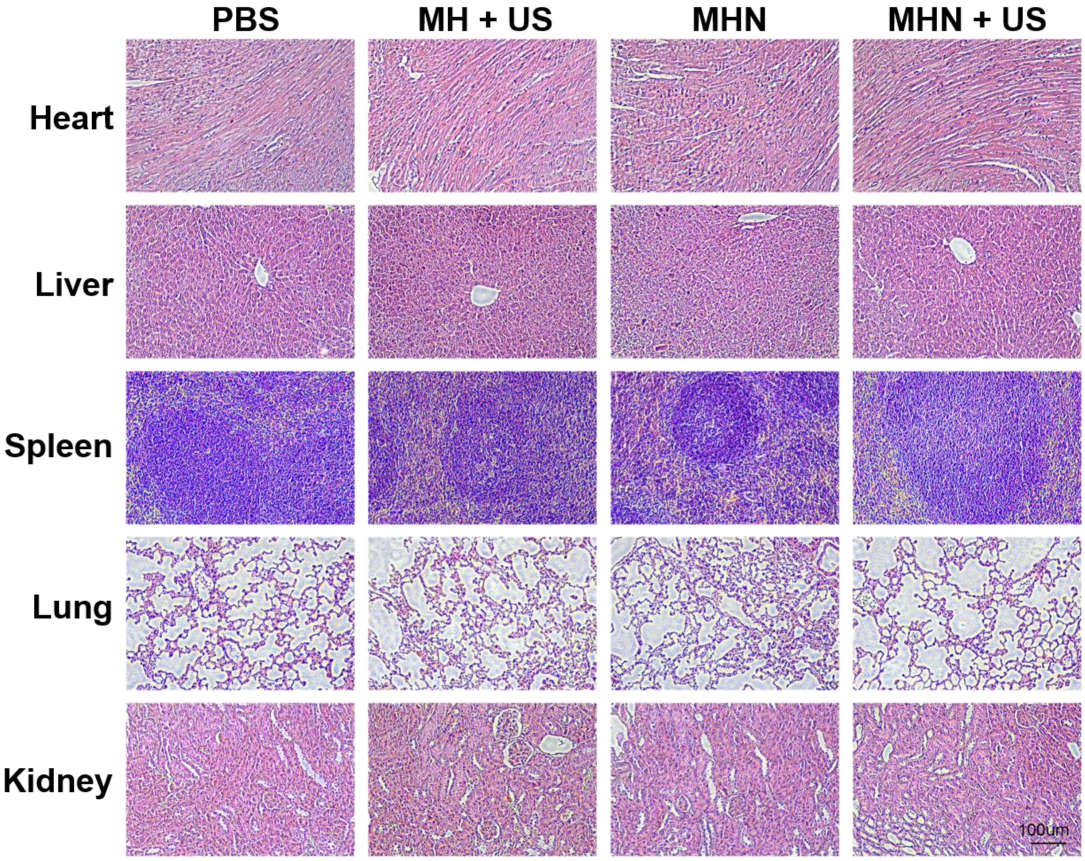

Figure S17 HE staining of major tissue slices (heart, liver, spleen, lung and kidney) after treatment of different NGs, scale bar: 100 μm.

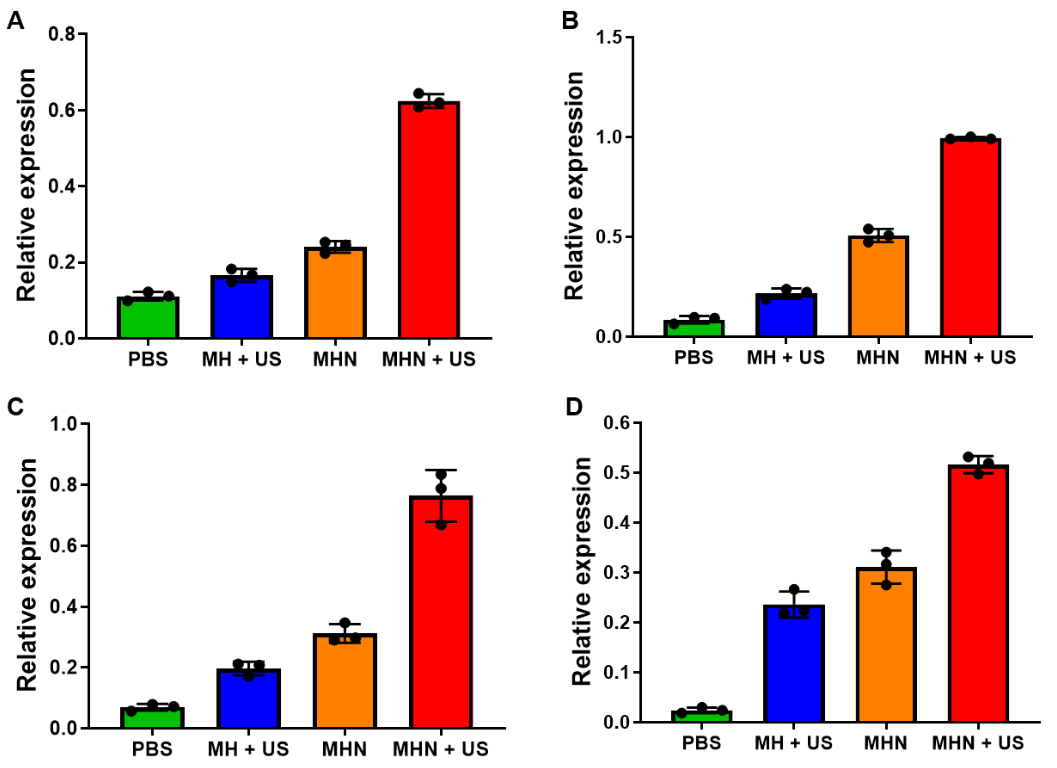

Figure S18 Quantitative data of western blot assay of pyroptosis-related proteins caspase 1 p20 (A), GSDMD-NT (B) and cGAS-STING-related proteins p-STING (C), IRF3 (D) by extracted tumor cells (n = 3).
